# Antibacterial potency of acyclic diester, oleic acid, and *β*-amyrin tetradecanoate from *Acacia lahai* and *Leucas calostachys* against antibiotic-resistant bacteria

**DOI:** 10.3389/fmicb.2025.1604820

**Published:** 2025-06-25

**Authors:** Nicholas Kogo Kimutai, Philip A. Ogutu, Rahab Kamau, Charles Mutai

**Affiliations:** ^1^Department of Biological Sciences, Masinde Muliro University of Science and Technology, Kakamega, Kenya; ^2^Department of Pure and Applied Chemistry, Masinde Muliro University of Science and Technology, Kakamega, Kenya; ^3^Department of Medical Laboratory Science, Bomet University College, Bomet, Kenya

**Keywords:** resistance, *Acacia lahai*, fatty acids, *Leucas calostachys*, acyclic diester and traditional medicine

## Abstract

**Introduction:**

The increase in antibiotic-resistant microorganisms has led to the search of new and effective antimicrobial agents. Natural products from plants may, therefore, serve as alternative sources of substances for the treatment of these infections. Traditional practitioners use concoctions of *Acacia lahai* and *Leucas calostachys* extracts for the treatment of wounds, skin diseases, coughs, ulcers, and intestinal ailments. This is because they are rich in potent antibacterial compounds. The objective of this study was to isolate bioactive compounds from *A. lahai* and *L. calostachys* using bioassay-guided fractionation.

**Methods:**

Bioactivity testing was performed against selected microbes using disc diffusion and broth microdilution methods, as recommended by the Clinical and Laboratory Standards Institute (CLSI). Pure compounds were isolated using chromatographic procedures, and their structures were elucidated based on 1D and 2D NMR analyses.

**Results:**

Fractionation yielded two fatty acids, namely, 5-(2, 5-dimethylhexyl) 1-isopentyl 3-hydroxy-2-methylpentanedioate (acyclic diester) (**1**) and *cis* oleic acid (**2**) from *A. lahai* and *L. calostachys*, respectively. In addition, *L. calostachys* yields terpenoid *β*-amyrin tetradecanoate (**3**). The three compounds were selectively active against the tested microorganisms, with minimum inhibitory concentrations (MICs) of 25.0 mg/mL and 100.0 mg/mL shown by *cis* oleic acid and *β*-amyrin tetradecanoate against *Staphylococcus aureus*, respectively. These two compounds were isolated and tested for antibacterial activity against this plant for the first time. In addition, an acyclic diester named 5-(2, 5-dimethylhexyl) 1-isopentyl 3-hydroxy-2-methylpentanedioate (**2**) was isolated and screened for antibacterial activity for the first time from the extracts of *Acacia lahai.*

**Conclusion:**

*Acacia lahai* and *Leucas calostachys* extracts and compounds showed antibacterial activity against multidrug-resistant bacteria. This study provides valuable insights into the development of effective antimicrobial agents.

## 1 Introduction

Antibiotic resistance of bacterial strains is a major problem in hospitals. The effectiveness of available antibiotics is uncertain due to genetic changes in microorganisms, including bacteria (World Health Organization, 2022). Concerns regarding antibiotic resistance are increasing, and new strategies for combating bacterial infections are needed (Kwon and Powderly, 2021). Antibiotic-resistant bacteria exhibit *in vitro* resistance to more than one antimicrobial agent in three or more classes. Examples of antibiotic-resistant bacteria include ESBL (extended-spectrum beta-lactamase) *Escherichia coli* and methicillin-resistant *Staphylococcus aureus.* ESBL *Escherichia coli* is a strain of *Escherichia coli* that contains resistant genes to ESBL-positive drugs such as penicillin, cephalosporin, and aztreonam (Kwon and Powderly, 2021). Fatty acids have long been known for their antimicrobial activity and are considered possible alternatives or complements to classical antibiotics (Yoon et al., 2018). For instance, fatty acids have been found to prevent *Escherichia coli* from forming characteristic lesions on epithelial cells in intestinal walls (Ellermann et al., 2021). Long-chain fatty acids interact with sensory proteins and transcriptional regulators that regulate the expression of infection-related genes. Consequently, long-chain fatty acids may disarm bacterial pathogens from their virulence factors (Caroline et al., 2023).

Methicillin-resistant *Staphylococcus aureus (MRSA)* is a strain of *Staphylococcus aureus* that has developed resistance to *β*-lactam antibiotics. This bacterium is responsible for several difficult-to-treat infections in humans (Timothy and Foster, 2017). The rapid development of microbial resistance to conventional medicine has necessitated the exploration of alternative medicines available in medicinal plants, such as *Acacia lahai* and *Leucas calostachys.*

*Acacia lahai* is a flat-topped tree with grey bark belonging to the family Fabaceae, which is widely distributed in Kenya. This plant is used for the treatment of coughs and pneumonia in humans (Kimutai et al., 2019). Before this study, the antibacterial activities of methanol extracts from the stem bark of *A. lahai* were active against *S. aureus,* with an inhibition zone of 18.0 mm (Kimutai et al., 2020), which was attributed to the chemical compounds present (Hannaa et al., 2022). However, further fractionation and purification were performed; therefore, very little phytochemical investigation has been conducted on this plant. Phytochemical screening of the bark of Acacia species, such as *Acacia nilotica* and *Acacia dealbata,* revealed the presence of tannins, fatty acids, and terpenoids (Yang et al., 2020). Moreover, saturated fatty acids are found in higher amounts than unsaturated fatty acids in the leaves and bark of *Acacia dealbata* (Oliveira et al., 2015). In addition, a new straight-chain fatty acid was isolated from *Acacia nilotica* (Chaubal et al., 2006). The use of these plants in traditional medicine for the treatment of bacterial infections is associated with the presence of fatty acids. Fatty acids have been found to downregulate many genes involved in adaptation to stressful conditions, especially in the gastrointestinal tract, including acid, bile, and osmotic stress (Chen et al., 2021).

*Leucas calostachys* is a shrubby plant with hairy stems and sessile leaves, and it grows 1-2 m tall. In many parts of Kenya, it is traditionally used to treat wound infections, typhoid, and cough (Keita et al., 2022). Phytochemical screening of chloroform leaf extracts has shown the presence of terpenoids, alkaloids, and phenols (Jeruto et al., 2011). The antimicrobial activities of leaf methanol extracts were active against *P. aeruginosa* and *Candida albicans* with inhibition zones of 11.00 mm and 13.0 mm, respectively. Although considered understudied, the extracts have good activity against microorganisms (Schultz et al., 2021). Studies conducted on the root hexane extract of *L. calostachys* showed activity against selected bacteria, with the highest inhibition zone (12.00 mm) against *E. faecalis* (Kimutai et al., 2021).

A recent study reported the bioactivity of crude solvent extracts and fractions from *L. calostachys,* (Kimutai et al., 2021). In many plants, pure compounds are responsible for various pharmacological properties, including antibacterial activity (Keita et al., 2022). The discovery of new plant species and the functional explanation of their bioactive molecules are the main goals of continuous research in phytochemical science (Schultz et al., 2021). They are a source for the development of new chemotherapeutic medications for the treatment of bacterial infections (Hejja et al., 2024). This study reports the antibacterial activities of extracts, fractions, and isolated compounds from the stem bark of *A. lahai* and the root bark of *L. calostachys.* This study provides a scientific basis for the use of these plants in traditional medicine for the development of phytomedicines.

## 2 Materials and methods

### 2.1 Plant materials

The stem bark of *A. lahai* and the root bark of *L. calostachys* were collected during December 2017 in Nandi County. It was identified by Dr. Bernard Wanjohi at the University of Eldoret. The voucher specimens, labeled KN/Ndi/17/05/010 for *A. lahai* and KN/Ndi/17/05/028 for *L. calostachys*, were deposited at the same institute.

### 2.2 Initial extraction of plant material

Identification of active plant constituents began with an antibacterial test using crude extracts (Zhang and Cheng, 2022). A sample of powdered bark, whole roots, and leaves of the respective plants, weighing 50 grams each, was exhaustively extracted by soaking in methanol. The extraction was carried out in a 250 mL conical flask, with 200 mL of the respective solvent added. The extracts were allowed to stand for 24 h at room temperature and then filtered through Whatman No. 1 filter paper. The filtrates were concentrated using a rotary evaporator and air-dried for 3 days. The extracts were placed in sterile airtight vials, weighed, and kept in a desiccator at readiness for use (Eftekhari et al., 2017).

### 2.3 Successive extractions of the plant material

Successive extractions were performed by dissolving the ground material (1 kg) in 2 L of hexane for 48 h. The soaked material was filtered, and the crude extracts were collected in a clean container. The crude extract was then concentrated using a rotary evaporator and left to dry in open air for 3 days, weighed, and stored in a desiccator at readiness for use. The residue, after extraction with hexane, was soaked in 2 L of dichloromethane for 48 h, and the extracts were filtered. The filtrate was concentrated, and the solvent was recovered by distillation using a rotary evaporator. The extract was concentrated, dried for 3 days, weighed, and stored in a desiccator. The residue, after extraction with dichloromethane, was dried and soaked in 2 L of ethyl acetate for 48 h, filtered, concentrated, dried, weighed, and stored in a desiccator. The residue after extraction with ethyl acetate was soaked in 2 L of methanol for 48 h, filtered, concentrated, dried, weighed, and stored in a desiccator. The extracts were then screened for antibacterial activity.

### 2.4 Isolation of compounds

#### 2.4.1 Acacia lahai

The ethyl acetate (EtOAc) extract from the stem bark of *A. lahai* was found to be active against the tested microorganisms and was, therefore, considered for the isolation of active compounds. The EtOAc stem bark extract (7.09 g) was adsorbed onto 10 g of silica gel and subjected to column chromatography on a silica gel column eluted with *n*-hexane/ EtOAc (95:5–5:95) followed by EtOAc/MeOH (99:1, 49:1). A total of 81 eluents were collected in 50 mL aliquots, and those with similar Rf values were pooled together to obtain 7 fractions labeled FA1-FA7. They were concentrated, left to dry, and tested for antibacterial activity. FA1 (106 g) showed higher antibacterial activity than the other tested samples (Table 1), and was therefore subjected to (10 g, SiO_2_) column chromatography eluted with (EtOAc/*n*-hexane) to yield one pure compound **1** (27.2 mg).

**Table 1:** Mean inhibition zones in millimeters of fractions of *A. lahai.*

Fractions	Mean inhibition zone diameters in millimeter for each *test bacteria*								
	MR*S.a*	*S.a*	*P.a*	*E.c*	E.*f*	*K.p*	*S.s*	*C.e*	*S.t*
FA1	13.93	14.73	11.43	6.00	12.10	8.00	7.00	13.03	6.00
FA2	6.00	6.00	6.00	6.00	6.00	6.00	6.86	10.80	6.00
FA3	6.00	6.00	6.00	6.00	6.00	6.00	6.00	6.00	6.00
FA4	10.00	11.12	6.00	6.00	6.00	9.11	11.66	6.00	6.00
FA5	6.00	6.00	6.00	6.00	6.00	6.00	6.00	6.00	6.00
FA6	6.00	6.00	6.00	6.00	6.00	7.71	10.10	6.00	6.00
FA7	6.00	6.00	6.00	6.00	6.00	6.00	6.00	6.00	6.00
+ cont.	21.0	23.0	23.0	21.0	21.0	18.0	20.0	21.0	22.0

Values are means of test replicates (*n* =3).Use the key in table. Key: F1-F7 (Fractions), + control (Gentamicin), MR.S. *a*- *Methicillin Resistant staphylococcus aureus*, *S. a*- *staphylococcus aureus*, *P. a- Pseudomonas aeruginosa*, *E. c-Escherichia coli*, *E. f- Enterococcus faecalis*, *K. p-Klebsiella pneumonia*, *S. s- Shigella sonnei*, *C. f-Citrobacter freudii and S.t-Salmonella typhi*.

#### 2.4.2 Leucas calostachys

The root bark hexane extract, which was bioactive, was used for the isolation of active compounds. The extract (6.07 g) was adsorbed onto 8.0 g of silica gel, subjected to column chromatography on a silica gel column, and eluted with *n*-hexane/ EtOAc (95:5–5:5) to obtain 39 eluents. Fractions with similar patterns on TLC were combined and labeled as FL1-FL5. They were further tested for antibacterial activities, and fraction FL4 was found to be the most active compared to the other fractions. The mixture was subjected to column chromatography using ethyl acetate and *n*-hexane as eluent. This process yielded a white oily compound **2** (97.2 mg) and a white solid compound **3** (38.4 mg).

### 2.5 Antimicrobial assays

Antibacterial activity was carried out at the Center for Microbiology Research (KEMRI) and Masinde Muliro University of Science and Technology (MMUST) using the disc-diffusion method.

The Gram-positive bacteria were *Staphylococcus aureus* ATCC 25923 and clinical isolate of methicillin-resistant *Staphylococcus aureus* (MR*SA*), and *Enterococcus faecalis,* while the Gram-negative bacteria were *Escherichia coli* ATCC 25922, ESBL *Escherichia coli, Pseudomonas aeruginosa* ATCC 27853, *Citrobacter freundii*, *Klebsiella pneumoniae*, *Salmonella typhi*, and *Shigella sonnei.*

The bacteria were cultured on Mueller-Hinton agar for 24 h at 37°C. A 0.5 McFarland standard suspension was prepared in normal saline. The suspension was spread uniformly on Muller–Hinton agar (MHA). A 6-mm sterile paper disc was impregnated with 10 μL of the test extracts, dried on a clean bench, and aseptically placed onto the surface of the inoculated media. The plates were then incubated at 37°C for 24 h. The zones of inhibition were measured as indicators of activity. Extracts with activity were fractionated and retested for their bioactivity. All tests were performed in triplicate. Gentamycin was used as a positive control, while dimethyl sulfoxide (DMSO) was used as a negative control. This study was performed following the recommendations of the National Committee for Clinical Laboratory Standards (CLSI) 1997.

#### 2.5.1 Disk diffusion assay

Antibacterial activities of the extracts, fractions, and compounds were determined using the paper disk diffusion method. Positive controls were set against the standard antibiotic gentamycin, whereas negative controls were set using a disk impregnated with solvent (DMSO).

#### 2.5.2 Minimum inhibitory concentration (MIC)

The MICs of the active plant extracts and compounds were determined using the broth microdilution method against the test microorganisms. This method is recommended by the National Committee for Clinical Laboratory Standards, now the Clinical Laboratory Standard Institute (CLSI) (NCCLS, 2002). The tests were performed in 96-well microtiter plates. Plant extracts and compounds were dissolved in the respective solvents and transferred into microtiter plates to make serial dilutions ranging from 10^1^, 10^2^, 10^3^….0.10.^10^ The final volume in each well was 100 μL. The wells were inoculated with 5 μL of microbial suspension and incubated at 37°C for 24 h. The MIC was recorded as the lowest extract concentration that demonstrated no visible growth compared to the control broth turbidity. Wells that were not inoculated with microbial suspensions served as controls. All assays were performed in triplicate, and the average values were computed and tabulated.

#### 2.5.3 Minimum bactericidal concentration (MBC)

The MBC was determined by collecting a loop full of broth from the wells that did not show any growth in the MIC assay, two wells above and two wells below the MIC value, and inoculating on sterile Muller–Hinton agar by streaking and incubating at 37°C for 24 h. The highest dilution that did not yield a colony fraction on solid medium was considered MBC.

### 2.6 Data analysis

The diameter of the inhibition zone around each disc was measured and recorded at the end of the incubation period. The average of the triplicate tests was calculated. The degree of activity of the extracts was expressed according to inhibition zone diameter as follows: no activity (<6 mm), active (7–11 mm), and very active (>12 mm).

## 3 Results

### 3.1 Isolated compounds

**Figure 1:**
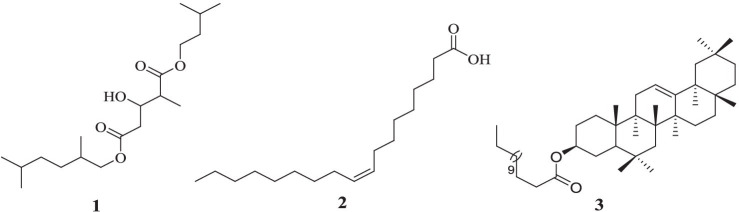
Structures of compound **1** from *A. lahai* and compounds **2** and **3** from *L. calostachys.* The structures of these compounds were identified by interpretation of the ^I^H- NMR, ^13^C- NMR, ^1^H -^1^H COSY and ^1^H-^13^C HMBC data shown below and by comparison with literature values as follows: The structure of compound **1** was identified as 5-(2, 5-dimethylhexyl) 1-isopentyl 3-hydroxy-2-methyl pentanedioate (Chaubal et al., 2006). Compound **2** was identified as *oleic acid* (Wekesa, 2014), whereas compound **3** was elucidated as *β*-amyrin tetradecanoate (Machumi, 2011).

*Acacia lahai* bark extract yielded 5-(2,5-dimethylhexyl) 1-isopentyl 3-hydroxy-2-methylpentanedioate (**1**) (acyclic diester). The antibacterial activity-guided fractionation of *Leucas calostachys* root extracts (Kimutai et al., 2021) led to the isolation of two known compounds, namely oleic acid (**2**) and *β*-amyrin tetradecanoate (**3**) (Figure 1).

### 3.2 Physical and spectroscopic data of the isolated compounds

#### 3.2.1 [5-(2, 5-dimethylhexyl) 1-isopentyl 3-hydroxy-2-methylpentanedioate] (**1**)

White powder (27.2 mg) soluble in methanol,^1^HNMR (400 MHz, CD_3_OD): *δ*1.96 (*m,* H-2), δ 3.53 (*d*, *J* = 3.6, H-3), *δ* 1.62 (*m,* H-4), δ 1.78 (*m,* H-5), *δ* 1.04 (*d*, *J* = 2.4, H-6), *δ* 3.45 (*d*, *J* = 4.4, H-1′), δ 2.28 (*m,* H-2′), *δ* 1.28 (*m,* H-3′), δ 1.62 (*m,* H-4′), δ 1.28(*m,* H-5′), *δ* 0.97 (*s,* H-6′), *δ* 1.02 (*d*, *J* = 2.0, H-7′), *δ* 1.06 (*d*, *J* = 7.2, H-8′), *δ* 3.59 (*dd*, *J* = 5.2, *J* = 6.4, H-1′′), *δ* 1.62 (*m,* H-2′′), *δ* 1.78 (*m,* H-3′′), *δ* 0.99 (*d*, *J* = 1.2, H-4′′) and *δ* 1.00 (*d*, *J* = 3.2, H-5′′). ^13^C NMR (400 MHz, CD_3_OD): *δ* 174.9(C1), δ 37.8(C2), δ 60.8(C3), δ 41.8(C4), δ 173.4(C5), δ 15.7(C6), δ 61.7(C1′), δ 30.9(C2′), *δ* 26.2(C3′), δ 30.9(C4′), *δ* 25.9(C5′), δ 12.4(C6′), *δ* 17.9(C7′), δ 19.2(C8′), *δ* 54.6(C1′′), *δ* 41.8(C2′′), *δ* 26.2(C3′′), *δ* 22.2(C4′′) and *δ* 23.4(C5′′).

#### 3.2.2 [*Cis* oleic acid] (**2**)

White oily solid (97.2 mg) soluble in hexane, ^1^H NMR (600 MHz, CDCl_3_): *δ* 2.34, *t*, (*J* = 5.5, H-2), *δ* 1.64, t, (*J* = 5.5, H-3), *δ* 2.15, (*m,* H-4), δ 1.29 (*m,* H-5), δ 1.29 (*m,* H-6), *δ* 1.26 (*m,* H-7), *δ* 1.29 (*m,* H-8), *δ* 5.35, *d* (J = 6.0, H-9), *δ* 5.34, *d* (J = 6.0, H-10), *δ* 2.01 (*m,* H-11), *δ* 1.29 (*m* H-12), *δ* 1.29 (*m,* H-13), *δ* 1.26 (*m,* H-14), *δ* 1.29 (*m,* H-15), *δ* 1.28 (*m,* H-16), and *δ* 0.86, (*t, J =* 6.0, H-17). ^13^C NMR(600 MHz, CDCl_3_): *δ* 180.3(C1), δ 34.3(C2), δ 24.8(C3), δ 27.3(C4), δ 29.3(C5), δ 29.2(C6), δ 29.7(C7), *δ* 29.2(C8), δ 130.2(C9), δ 129.9(C10), δ 29.3(C11), *δ* 29.7(C12), *δ* 29.4(C13), δ 29.6(C14), δ 29.3(C15), *δ* 29.2(C16), *δ* 29.9(C17) and *δ* 14.3(C18).

#### 3.2.3 [*α*-amyrin tetracosanoate] (**3**)

White solid (38.1 mg) soluble in ethyl acetate, ^1^HNMR (400 MHz, CD_2_Cl_2_): *δ* 1.63, (*m,* H-1), 1.08 (*m,* H-1), 1.62 (*m,* H-2), *δ* 1.90 (*m, H*-2), δ 4. 45 (*dd*, J = 8.0, J = 4.0, H-3), *δ* 0. 85 (*m, H*-5), *δ* 1.45 (*m,* H-6), *δ* 1.54 (*m, H*-7), *δ* 1.33(*m, H*-7), *δ* 1.45 (*m, H*-7), *δ* 1.61 (*m, H*-9), *δ* 1.88 (*m,* H-11), *δ* 5.19 (*t*, J = 4.0, H-12), *δ* 1.78 (*m,* H-15), *δ* 0.95 (*m,* H-15), *δ* 2.02 (*m, H*-16) *δ* 0.79 (*m,* H-16), 1.95 (*dd*, J = 8.0, H-18), *δ* 1.69 (*m, H*-19), δ 1.02 (*m, H*-19), δ 1.35 (*m,* H-21), *δ* 1.10 (*m,* H-21), δ 1.38 (*m,* H-22), δ 1.34 (*m,* H-22), *δ* 0.86 (*s,* H-23), *δ* 0.84 (*s,* H-24), *δ* 0.97 (*s,* H-25), *δ* 0.98 (*s,* H-26), *δ* 1.14 (*s,* H-26), *δ* 0.83 (*s,* H*-*28) *δ* 0.88 (*s,* H-29), *δ* 0.87 (*s,* H-30), 1.53(*m*, H-2′), 1.55(*m*, H-3′), 1.21–1.34(m, H-4′- 12′), 1.31(m, H-13′), and *δ* 0.75 (*t*, J = 8.0, H-14′). ^13^C NMR (400 MHz, CD_2_Cl_2_): *δ* 38.1(C1), *δ* 23.8(C2), *δ* 81.1(C3), *δ* 38.1(C4), *δ* 55.8(C5), *δ* 18.5(C6), *δ* 31.5(C7), *δ* 40.2(C8), *δ* 47.9(C9), *δ* 37.2(C10), δ 24.1(C11), δ 122.1(C12), δ 145.3(C13), δ 42.1(C14), *δ* 26.1(C15), δ 27.3(C16), δ 32.8(C17), δ 47.6(C18), δ 47.1(C19), δ 31.3(C20), δ 35.1(C21), δ 37.5(C22), δ 28.1(C23), δ 16.6(C24), δ 15.7(C25), δ 16.9(C26), δ 26.0(C27), δ 28.6(C28), δ 33.5(C29), δ 23.8(C30), δ 171.0(C1′), 34.9(C2′), 23.9(C3′), 26.5–34.5(C4′-12′),32.6(C13′) and δ 14.7(C14′).

### 3.3 Antibacterial activities

The variation in the antibacterial activities of the fractions and extracts of *Acacia lahai* was due to the distribution of the active constituents within the various fractions.

Minimum inhibitory concentration (MIC) and Minimum bactericidal concentrations (MBC) of the active isolated compounds was determine to understand the mode of action.

## 4 Discussion

The antimicrobial activities of the initial methanol crude extracts of *A. lahai* were higher than those of the successive fractions and isolated compounds **1**. The highest inhibition zone (18.33 mm) was recorded for MR. *S. aureus* (Table 2). Interestingly, the extracts were potent against antibiotic-resistant bacteria with inhibition zones of 18.33 mm and 8.0 mm against MR. *S. aureus* and ESBL *E. coli,* respectively. This finding is in agreement with the results reported by Larson et al. (2016) and Cushine et al. (2020). A plausible reason for this is that methanol might have extracted compounds that are active against bacteria (Saavedra et al., 2017). All the successive extracts were more active against the selected Gram-positive bacteria than Gram-negative bacteria, with an inhibition zone of 7.33–11.20 mm (Table 2). This could be because Gram-negative bacteria have an additional outer membrane comprising a highly hydrophilic lipopolysaccharide layer (Touche et al., 2023). The ethyl acetate solvent extracts were slightly more active than other extracts. Therefore, the authors considered the extraction of antimicrobial compounds, as explained in previous studies (Ambarwati et al., 2017).

**Table 2:** Inhibition zones in millimeters of crude and successive extracts of *A. lahai.*

Extracts	MR-*S.a*	Average inhibition zone diameters in millimeters for each test microorganism							
		*S. a*	*P. a*	*E. c*	E. *f*	*S.s*	*K. p*	*C. f*	S*.t*
Crude	18.33	15.30	20.20	6.00	10.10	6.00	8.00	7.00	6.00
H	9.07	7.97	6.00	6.00	6.00	6.00	6.00	9.23	6.00
D	9.00	9.63	8.10	6.9	8.10	6.00	6.00	6.00	6.00
E	9.93	11.20	8.37	6.00	6.00	6.00	6.00	6.00	6.00
M	8.48	8.13	6.00	7.33	6.00	6.00	6.00	6.00	6.00
+ cont.	21.0	23.0	23.00	21.00	21.00	20.00	18.00	21.00	22.17

Values represent the mean of the test replicates (*n* = 3). Key: Crude—total methanol extract, H—Hexane, D—Dichloromethane, E—Ethyl acetate, and M—Methanol. Key: + control (gentamicin), MR.*S. a*—Methicillin-Resistant *Staphylococcus aureus*, *S. a*—*Staphylococcus aureus*, *P. a*—*Pseudomonas aeruginosa*, *E. f*—*Enterococcus faecalis*, *S. s*—*Shigella sonnei*, *K. p*—*Klebsiella pneumonia*, *E.c*—*Escherichia coli*, *E. E.c*—ESBL *Escherichia coli*, S.t—*Salmonella typhi*, and *C. f*—*Citrobacter freudii*.

**Figure 2:**
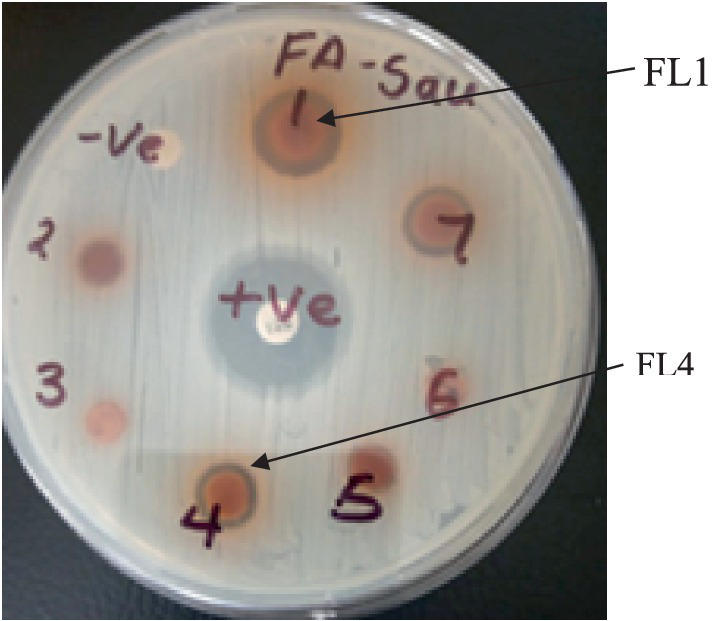
Plate showing the inhibition zones of fractions 1 and 4 of *Acacia lahai.*

**Figure 3:**
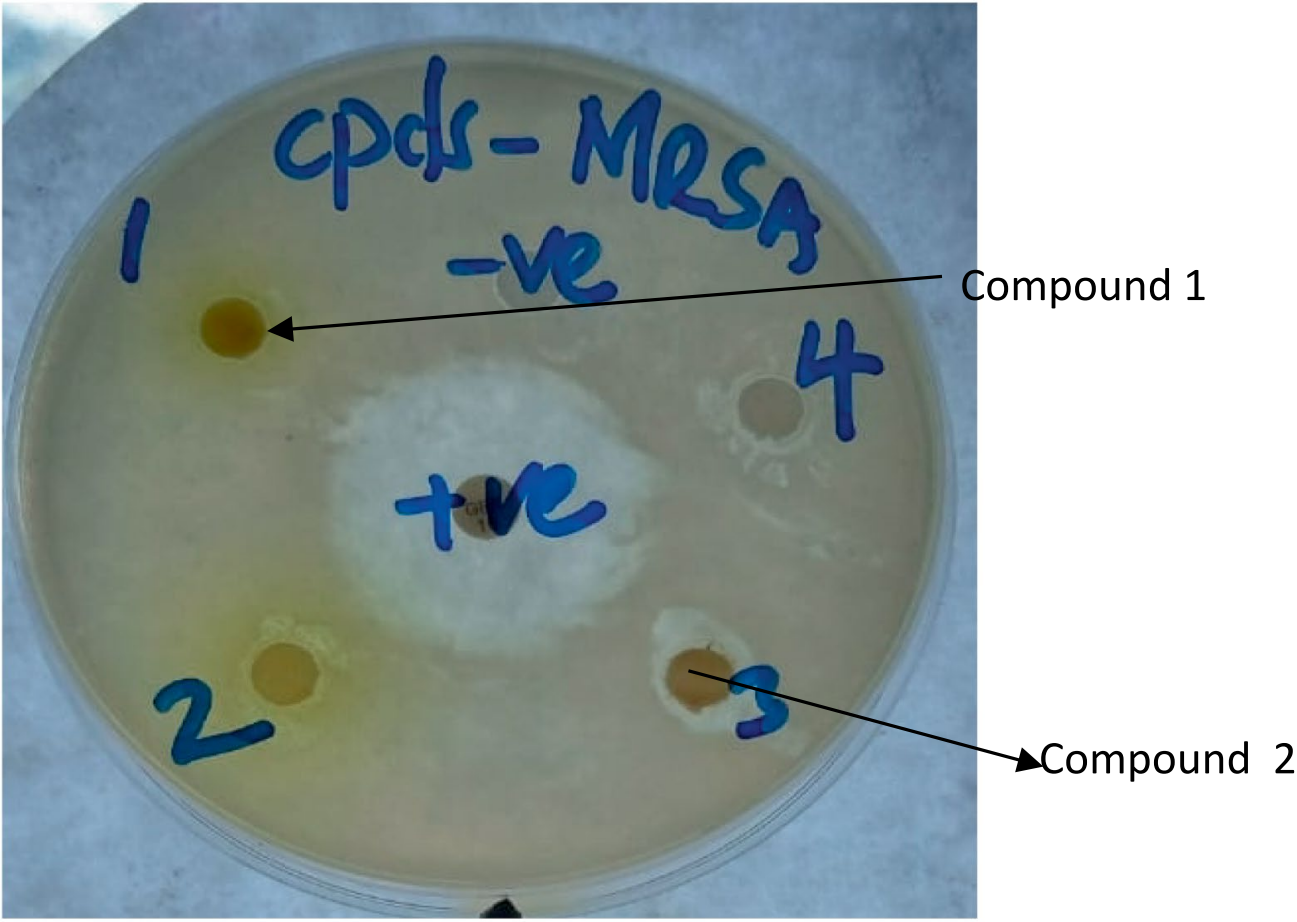
Plate showing inhibition zones of compounds 1 and 2 against MR-*S. aureus.* (The numbers written on the plate with a marker pen are serial numbers).

The differences in the activities of the extracted fractions of *A. lahai* can be attributed to the distribution of active constituents within various fractions. This is in agreement with the methanol extracts of *M. oleifera* leaves, which showed different inhibition patterns against different bacterial strains (Muhuha et al., 2018). This makes it possible to select the fraction with the highest zone of inhibition for purification to obtain pure compounds. For instance, fractions from *A. lahai-*coded FA1 had significant activity against five bacteria, namely MR. *S. aureus*, *S. aureus*, *P. aeruginosa*, *E. faecalis,* and *C. freudii* with inhibition zones of 13.93, 14.73, 11.43, 12.10, and 13.03 mm, respectively (Table 1 and Figure 2). Hence, this fraction was subjected to purification to obtain compound 5-(2, 5-dimethylhexyl) 1-isopentyl 3-hydroxy-2-methyl pentanedioate (World Health Organization, 2022). Compound **1** showed good activity against *S. aureus,* with an inhibition zone of 8.86 mm (Table 3 and Figure 3). The decreased activity observed for pure compound **1** could be due to the loss of synergy between the phytochemicals responsible for the bioactivities shown by the fractions and crude extract. In addition, compound **1** was a saturated fatty acid with moderate activity when incorporated into bacterial cell membranes (Mitchell and Ellermann, 2022).

**Table 3:** Mean inhibition zone diameters in millimeters of isolated compounds against selected bacteria.

S. No.	Compounds	Mean inhibition zone diameters in millimeters for each test bacterium								
		MR*S. a*	*S. a*	*P. a*	*E. c*	E. *f*	E. *E.c*	*K. p*	*C. f*	*S.t*
1	**1**	8.86	6.00	6.00	7.90	10.10	7.73	6.00	7.93	6.00
3	**2**	10.83	10.43	9.87	6.70	6.00	7.23	6.00	6.00	6. 76
2	**3**	8.86	10.16	6.00	6.00	6.00	6.00	6.90	6. 70	6.00
	+ cont.	21.20	23.17	23.03	21.13	21.23	22.17	18.90	21.13	22.96

Values represent the mean of the test replicates (*n* = 3). Use the key in Table 1.

*Enterococcus faecalis* was the least susceptible Gram-positive bacterium with low inhibition zones on most fractions; this could be due to resistant genes developed by clinical isolates when they colonize the intestines for a long time (Farman et al., 2019) (Table 1). Fatty acids such as compounds **1** and **2** have been found to regulate genes in the intestinal tract (Henderson et al., 2020). The most susceptible Gram-negative bacteria were *C. freudii,* with FA1, FA2, and FA4 having inhibition zones of 13.03 mm, 10.80 mm, and 11.66 mm against the same organisms, respectively. Fractions FA1 and FA7 were also active against *P. aeruginosa* with inhibition zones of 11.43 mm and 10.73 mm, respectively. The other Gram-negative bacteria were susceptible to only one fraction (Table 1) because of their morphology (Yoon et al., 2018).

Previous studies showed that crude hexane extracts of *L. calostachys* were more active than both successive extracts and fractions against both Gram-negative and Gram-positive bacteria (Kimutai et al., 2021). MBC of 3.15 mg/mL was obtained from total methanol extracts against methicillin-resistant *Staphylococcus aureus* (Kimutai et al., 2021). This supports the hypothesis that compounds in plants, in many cases, act synergistically (Sanhueza et al., 2017). However, in some cases, compounds can be antagonistic to each other, leading to low activity (Paolo et al., 2014). *L. calostachys* hexane extracts were bioactive and were fractionated. This yielded two compounds: one white oily compound **2** and a white solid compound **3.** *Cis* oleic acid (**2**) and *β*-amyrin tetradecanoate (**3**) were obtained almost simultaneously from successive eluents from *L. calostachys* hexane extracts. Esters, such as tetracosanoate, form part of the terpenoid and are mixed with other fatty acids, such as oleic acid. This usually makes it difficult to distinguish and separate them, and they have only been previously isolated as mixtures (Mafezoli et al., 2024).

Esters are connected to triterpenoids at carbon C-3 (Yue et al., 2015). In this study, oleic acid (**2**) and *β*-amyrin tetradecanoate (**3**) were isolated as separate compounds, elucidated, and tested for their antimicrobial activity. Terpenoids such as *β*-amyrin are found in many plants and have been isolated from *Leucas aspera* (Alam et al., 2014). Compounds **2** and **3** were isolated and tested from this plant for the first time. In addition, an acyclic diester named 5-(2, 5-dimethylhexyl) 1-isopentyl 3-hydroxy-2-methylpentanedioate (Kwon and Powderly, 2021) was also isolated for the first time from the ethyl acetate extract of *Acacia lahai.* However, esters have also been isolated from Acacia species before. For instance, a straight-chain diester named pentacosane dioic acid dihexadecyl ester was isolated from *Acacia nilotica* (Chaubal et al., 2006). In addition, straight-chain fatty acids, heptacosane-1, 2, 3-triol, and pentacosane dioic acid were isolated from *Acacia nilotica* (Oliveira et al., 2020).

The antibacterial activities of the three compounds had promising results; *cis* oleic acid (**2**) and *β*-amyrin tetradecanoate (**3**) recorded inhibition zones of 10.43 and 10.16 mm against *S. aureus,* respectively (Table 3). Both compound 5-(2, 5-dimethylhexyl) 1-isopentyl 3-hydroxy-2-methylpentanedioate (**1**) and oleic acid (**2**) had an inhibition zone of 8.86 mm against methicillin-resistant *S. aureus* (Table 3 and Figure 3). This is possibly because fatty acids are known to destroy the cell membrane of *S. aureus* (Tan et al., 2024). However, there were observed differences in the inhibition zones against the other bacteria. For instance, *cis oleic* acid (Kwon and Powderly, 2021) had an average inhibition zone of 7.86 mm against all the bacteria tested, whereas 5-(2, 5-dimethylhexyl)-1-isopentyl 3-hydroxy-2-methylpentanedioate (**1**) had an average of 7.39 mm. The difference in their activities may be due to their structural differences: oleic acid (**2**) is an unsaturated fatty acid, and 5-(2,5-dimethylhexyl) 1-isopentyl 3-hydroxy-2-methylpentanedioate (**3**) is a saturated fatty acid. In addition, compound **2** had an MIC of 25.0 and 6.250 mg/mL against *S. aureus* and MR. *S. aureus,* respectively (Table 4). This is due to the fact that unsaturated fatty acids are more active against bacteria than saturated fatty acids due to their ability to penetrate the cell membrane of bacteria, causing lysis or growth inhibition (Desbois et al., 2008). Another reason is the steric effect; oleic acid has *cis-*type double bonds and is adsorbed in the cell membrane easily due to its bent structure (Desbois et al., 2008). Fatty acids in the skin, such as oleic acid, are considered to be potential antimicrobials (Fischer, 2020). Fatty acids are used as antimicrobial agents by many organisms to protect themselves against microbial pathogens. The human skin, respiratory tract, and gastrointestinal tract represent three major environments in which bacterial pathogens encounter antimicrobial fatty acids produced by the host or resident microbiota (Metzler-Zebeli et al., 2021).

**Table 4:** MIC and MBC of the active compounds against susceptible bacteria.

S. No.	MIC and MBC in mg/ml for the active compounds against sensitive bacteria				
	Compounds	*S. aureus* MIC	MBC	MR. *S. aureus* MIC	MBC
3	**2**	25.00 ± 0.0	50.00 ± 0.0	6.25 ± 0.00	25.00 ± 0.0
2	**3**	100.00 ± 0.0	–	Nd	Nd
+ ve	Gent.	12.50 ± 0.00	12.50	±1.56	±3.125
– ve	DMSO	–	–	–	–

Values represent the mean of the test replicates ± standard deviation (*n* = 2). This was performed in duplicate because of the small quantities of the compounds. Key: –ve control (DMSO—dimethyl sulphoxide), +ve control (gentamicin), − No activity (static), ND—Not determined, *MR. S. a*—Methicillin-Resistant *Staphylococcus aureus*, *S. a*—*Staphylococcus aureus.*

The mode of action of the active isolated compounds was determined using the broth microdilution method. Compound **2** was bactericidal against MR. *S. aureus* and *S. aureus* with MBCs of 25.0 and 50.0 mg/mL, respectively (Table 4). However, compound **3** was bacteriostatic against *S. aureus,* as indicated by its growth at all the concentrations tested, despite having an MIC of 100.0 mg/mL. This compound might have targeted protein synthesis (Metzler-Zebeli et al., 2021).

Medium- and long-chain fatty acids act as signaling compounds that control important traits of bacterial pathogens, such as biofilm formation and virulence (Mitchell and Ellermann, 2022). Fatty acids also modulate oxidative stress and inflammatory responses in tissues by regulating the transcription factors NF-Kb and PPAR-*α*. For instance, docosahexaenoic acid modulates lipid metabolism and antioxidant hydroxyl tyrosol. This diminishes the oxidative stress underlying fatty liver, which induces steatosis (Soto-Alarcon et al., 2024). Obesity induced by a high-fat diet, which is associated with liver steatosis and oxidative stress, can be eliminated by co-administration of docosahexaenoic acid and hydroxyl tyrosol (Ortiz et al., 2020; Mirzaei et al., 2022). Moreover, dietary fat affects the composition and function of the natural microbial inhabitants of the gut, and gut microbes contribute to the overall fatty acid metabolism in the gastrointestinal tract (Chadaideh and Carmody, 2021; Thomasen et al., 2022). Not surprisingly, bacteria are capable of developing resistance to the antimicrobial activity of fatty acids. Fatty acid-tolerant strains have been isolated in broth containing increasing concentrations of antimicrobial medium- and long-chain fatty acids (Thomasen et al., 2022).

Compound **3**, *β*-amyrin tetradecanoate, was also active against MR. *S. aureus* and *S. aureus,* with inhibition zones of 8.86 and 10.16 mm, respectively (Table 3). This compound is a terpenoid that has the ability to disrupt microbial membranes. This is because of the presence of a hydroxyl group at carbon 3, which acts as an efficient uncoupler of the bacterial plasma membrane, causing lysis and death (Yang et al., 2020). However, the isolated terpenoid lacked a hydroxyl group at carbon C-3; instead, it was attached to an ester. This may explain the antibacterial activity observed. Studies on oleanolic acid derivatives have shown that they are specific HIV-1 inhibitors, making them potential candidates for anti-HIV drugs (Yonglin et al., 2020).

## 5 Conclusion

In this study, the isolated compounds exhibited antibacterial activity against both Gram-positive and Gram-negative bacteria, thereby demonstrating their potential as natural antibacterial agents. However, further studies are required to evaluate the *in vivo* and synergistic effects of these compounds and their extracts in combination with existing antibiotics to enhance their antimicrobial efficacy. Overall, this study contributes to the growing field focusing on discovering antimicrobial agents that can combat antibiotic-resistant bacteria. This study provides valuable insights into the development of effective antimicrobial agents and supports the use of decoctions from these plants in traditional medicine for treating infectious diseases caused by bacteria.

## Data Availability

The original contributions presented in the study are included in the article/supplementary material, further inquiries can be directed to the corresponding author/s.
