# Supplementary material for: Antibacterial potency of acyclic diester, oleic acid, and β-amyrin tetradecanoate from Acacia lahai and Leucas calostachys against antibiotic-resistant bacteria
Source: Front Microbiol. 2025 Jun 25;16:1604820. doi: 10.3389/fmicb.2025.1604820 (PMC12237972; doi:10.3389/fmicb.2025.1604820)
Supplement: Supplementary file 1 [file Table_1.docx]

**^I^H-, ^13^C- NMR data of compound 2 in CDCl_3_ (400MHz) and compound 3 in CD_2_Cl_2_), (400 MHz).**

| C/No. | **Compound 2**  Cδ(ppm) | Hδ (ppm)  (J in Hz) | **Compound 3**  Cδ (ppm) | Hδ (ppm)  (J in Hz) |
| --- | --- | --- | --- | --- |
| 1 | 38.1 | 1.63, (*m*),1.08 (*m*) | 180.3 | - |
| 2 | 21.4 | 1.62(*m*),1.90 (*m,*) | 34.3 | 2.34 (*t*,7.5, 5.5) |
| 3 | 81.1 | 4.45 (*dd*, 8.0,4.0) | 24.8 | 1.64 (*t*,7.5, 5.5) |
| 4 | 38.0 | **-** | 27.3 | 2.15(*m)* |
| 5 | 55.8 | 0.85(*m)* | 29.3 | 1.29 (*m*) |
| 6 | 18.5 | 1.45 (*m*),1.54 (*m)* | 29.2 | 1.29 (*m*) |
| 7 | 31.5 | 1.33(*m*),1.45(*m*) | 29.7 | 1.26 (*m)* |
| 8 | 40.2 | - | 29.2 | 1.29 (*m*) |
| 9 | 47.9 | 1.61(*m*) | 130.2 | 5.35(*d* 6.0,3.0) |
| 10 | 37.2 | - | 129.9 | 5.34 (*d* 6.0,3.0) |
| 11 | 24.1 | 1.88 (*m*),1.61 (*m*) | 29.3 | 2.01 (*m*) |
| 12 | 122.1 | 5.19 (*t* ,4.0) | 29.7 | 1.29 (*m*) |
| 13 | 145.3 | - | 29.4 | 1.29 (*m*) |
| 14 | 42.1 | - | 29.6 | 1.29 (*m*) |
| 15 | 26.1 | 1.78 ( *m*), 0.95 (m) | 29.3 | 1.26 (*m)* |
| 16 | 27.3 | 2.02 (*m*) 0.79 (*m*) | 29.2 | 1.29 (*m*) |
| 17 | 32.8 | - | 29.9 | 1.28 (*m)* |
| 18 | 47.6 | 1.95(*dd ,* 8.0 ,4.0) | 14.3 | 0.86 (*t,* 6.0,3.0,1.8) |
| 19 | 47.1 | 1.69 ( *m*),1.02( *m*) |  |  |
| 20 | 31.3 | - |  |  |
| 21 | 35.1 | 1.35 ( *m*),1.10( *m*) |  |  |
| 22 | 37.5 | 1.38 (*m*),1.34 ( *m*) |  |  |
| 23 | 28.1 | 0.86 (*s*) |  |  |
| 24 | 16.6 | 0.84 (*s*) |  |  |
| 25 | 15.7 | 0.97 (*s*) |  |  |
| 26 | 16.9 | 0.98 (*s*) |  |  |
| 27  28 | 25.4  28.5 | 1.14(*s*)  0.83 ( *s*) |  |  |
| 29  30 | 33.4  21.5 | 0.88( *s*)  0.87 (*s*) |  |  |
| 1′  2′  3′  4′-25′12′  26′/13′  27′-  28′14′ | 171.0  34.9  23.9  26.5-34.6  32.6  23.8  14.7 | -  1.53(*m*)  1.55(*m*)  1.21-1.42(*m*)  1.31(*m*)  1.32(*m*)  0.75, (*t,* 8.0,4.0,2.0) |  |  |
